# Microwave-assisted synthesis of ZnS@CuIn_x_S_y_ for photocatalytic degradation of coloured and non-coloured pollutants

**DOI:** 10.1038/s41598-024-66100-2

**Published:** 2024-07-12

**Authors:** Ashmalina Rahman, Fazlurrahman Khan, James Robert Jennings, Young-Mog Kim, Mohammad Mansoob Khan

**Affiliations:** 1 Chemical Sciences, Faculty of Science, Universiti Brunei Darussalam, Jalan Tungku Link, Gadong, BE 1410 Brunei Darussalam; 2 Institute of Fisheries Science, Pukyong National University, Busan, 48513 Republic of Korea; 3 Marine Integrated Biomedical Technology Center, The National Key Research Institutes in Universities, Pukyong National University, Busan, 48513 Republic of Korea; 4 Research Center for Marine Integrated Bionics Technology, Pukyong National University, Busan, 48513 Republic of Korea; 5 Applied Physics, Faculty of Science, Universiti Brunei Darussalam, Jalan Tungku Link, Gadong, BE 1410 Brunei Darussalam; 6 Optoelectronic Device Research Group, Universiti Brunei Darussalam, Jalan Tungku Link, Gadong, BE 1410 Brunei Darussalam; 7 Department of Food Science and Technology, Pukyong National University, Busan, 48513 Republic of Korea

**Keywords:** Copper indium sulfide, Copper sulfide, Zinc sulfide, CuInS_2_, CuS, ZnS, ZnS@CuIn_x_S_y_, Photocatalysis, Brilliant green, 4-nitrophenol, Chemistry, Materials science

## Abstract

Copper indium sulfide (CuInS_2_) exhibits strong visible light absorption and thus has the potential for good photocatalytic activity; however, rapid charge recombination limits its practical usage. An intriguing strategy to overcome this issue is to couple CuInS_2_ with another semiconductor to form a heterojunction, which can improve the charge carrier separation and, hence, enhance the photocatalytic activity. In this study, photocatalysts comprising CuInS_2_ with a secondary CuS phase (termed CuIn_x_S_y_) and CuIn_x_S_y_ loaded with ZnS (termed ZnS@CuIn_x_S_y_) were synthesized via a microwave-assisted method. Structural and morphological characterization revealed that the ZnS@CuIn_x_S_y_ photocatalyst comprised tetragonal CuInS_2_ containing a secondary phase of hexagonal CuS, coupled with hexagonal ZnS. The effective band gap energy of CuIn_x_S_y_ was widened from 2.23 to 2.71 as the ZnS loading increased from 0 to 30%. The coupling of CuIn_x_S_y_ with ZnS leads to long-lived charge carriers and efficient visible-light harvesting properties, which in turn lead to a remarkably high activity for the photocatalytic degradation of brilliant green (95.6% in 5 h) and conversion of 4-nitrophenol to 4-nitrophenolate ions (95.4% in 5 h). The active species involved in these photocatalytic processes were evaluated using suitable trapping agents. Based on the obtained results, photocatalytic mechanisms are proposed that emphasize the importance of h^+^, O_2_^•–^, and OH^−^ in photocatalytic processes using ZnS@CuIn_x_S_y_.

## Introduction

Organic pollutants such as dyes are continuously being released into the environment as a result of increasing agricultural and industrial activities, posing major risks to environmental and public health. Brilliant green (BG) dye is a typical triphenylmethane dye with a complex structure that is stable and non-biodegradable. It is commonly used in biological staining, modern textiles, as well as in the paper, plastic, rubber, cosmetic, and antiseptic industries^1^. Apart from dyes, 4-nitrophenol (4-NP) is also a common toxic organic contaminant in water. 4-NP exhibits high toxicity and carcinogenicity as well as possessing high chemical and biological stability and water solubility^2^. As a result, removing organic pollutants including coloured and non-coloured pollutants is a major priority. Many conventional approaches including adsorption have been employed to address organic contaminants. However, there are several drawbacks including low efficiency, limited stability, and high cost^3^. These disadvantages result in the limited implementation of these techniques on an industrial scale. Semiconductor-based photocatalytic methods have gained substantial attention due to their promising applications in wastewater remediation^4,5^. Photocatalytic treatment of organic contaminants has the significant benefit of eliminating hazardous pollutants without leaving any harmful products or secondary pollutants^6–8^.

Amongst various semiconductors, CuInS_2_ is one of the most promising photocatalytic materials due to its narrow band gap energy of ~ 1.45 eV. CuInS_2_ exists in three different crystal structures including chalcopyrite, zinc blende and wurtzite^9^. CuInS_2_ has received increasing attention owing to its long-term stability against photocorrosion, appropriate band gap, and good solar conversion efficiency. It has been reported that it exhibited great potential in H_2_ evolution from water splitting^10,11^, photocatalytic degradation of organic pollutants^12^, photoelectrochemical reduction of CO_2_^13^, and photocatalytic reduction of Cr(VI)^14^. A range of synthetic approaches have been used to prepare CuInS_2_ with different structural compositions and morphology. Various synthetic approaches such as hot injection^15^, hydrothermal^16^, solvothermal^17^, microwave-assisted^18^, and sonochemical^19^ methods have been reported. Among these synthesis methods, microwave-assisted synthesis has several advantages in comparison to other more conventional approaches. Microwave assisted synthesis relies on efficient dielectric heating, primarily through two mechanisms, namely dipolar polarization and ionic conduction^20^. Dipolar polarization occurs when material with partial positive and negative charges aligns with the oscillating microwave field, inducing rotation and generating heat through friction. Ionic conduction involves completely dissolved charged particles oscillating under microwave irradiation leading to collisions with their neighboring molecules/atoms and subsequent heat production^21^. This method offers short reaction times as well as uniform and rapid heating, resulting in the formation of CuInS_2_ with high purity and a monodisperse particle size distribution^22,23^.

Zinc sulfide (ZnS) is another material that is of interest for photocatalytic applications owing to the highly negative reduction potential of photoexcited electrons in the ZnS conduction band in addition to being non-toxic, environmentally friendly, highly stable, and inexpensive^24^. However, ZnS suffers from some critical drawbacks that limit its photocatalytic efficiency. ZnS is only active under UV light irradiation owing to its wide band gap, which corresponds to just 5% of the total energy available from sunlight. ZnS also suffers from an intrinsically fast electron–hole recombination rate, which competes with interfacial charge transfer and thus limits the photocatalytic efficiency. Therefore, the fabrication of semiconducting heterostructures has become a promising trend and is likely to be the best alternative approach to overcome these drawbacks^25^. The formation of a heterojunction retains the basic features of the individual components while adding unique multifunctionalities such as enhanced charge separation and transfer of the photogenerated charge carriers between the different semiconductors^26–28^.

Recently, ZnS/CuInS_2_ heterostructures have drawn a lot of attention, however the usage of this composite in photocatalytic applications has not been fully explored^29–31^. The combination of CuInS_2_ and ZnS to create a junction shows the advantages of improving the separation and transfer of charge carriers and eventually decreasing the recombination rate of electron–hole pairs. The coupling of CuInS_2_ with ZnS to form a heterostructure is one of the promising approaches to enhance the photoactivity of both components and extend the photoresponse of the photocatalysts.

To date, the scope of photocatalytic applications of the ZnS@CuInS_2_ composites has been primarily limited to H_2_ generation, CO_2_ reduction, Cr(VI) reduction, and antibiotic and dye degradation^31–33^. However, most previously studied photocatalysts contained other active materials^30,34^ such as TiO_2_ and AgInS_2_ or were modified with mercaptopropionic acid and thioglycolic acid^35–37^.

In this work, CuInS_2_ with a secondary CuS phase (termed CuIn_x_S_y_) and CuIn_x_S_y_ loaded with ZnS (termed ZnS@CuIn_x_S_y_) were prepared via a microwave-assisted method. The effect of different percentages of ZnS on the structural, optical, and morphological properties of the CuIn_x_S_y_ was also studied. Moreover, by loading ZnS onto CuIn_x_S_y_, the recombination rate of photogenerated carriers was significantly suppressed and an enhancement in the photocatalytic degradation of coloured pollutant, BG, and the conversion of non-coloured pollutant, 4-NP, with a low photocatalyst dosage was achieved. A further in depth study of the active species responsible for the removal of these pollutants was also performed, providing insights into the underlying BG degradation and 4-NP conversion mechanisms. This work demonstrates that the promising ZnS@CuIn_x_S_y_ heterojunction photocatalyst has the potential for the complete elimination of organic pollutants in wastewater.

## Experimental section

### Chemicals

All reagents were used without further purification. For the synthesis, copper nitrate trihydrate (Cu(NO_3_)_2_·3H_2_O) and indium nitrate (In(NO_3_)_3_) were purchased from Alfa Aesar as the Cu and In sources, respectively. Zinc acetate dihydrate (Zn(CH_3_COO)_2_·2H_2_O), thiourea (CH_4_N_2_S), hydrogen peroxide (H_2_O_2_), and 4-NP (C_6_H_5_NO_3_) were purchased from Merck, Germany. The distilled water was purified using water still from Aquatron, England, and ethanol was purchased from Duksan Pure Chemicals Co. Ltd, South Korea. For the photocatalytic application tests, BG (C_27_H_34_N_2_O_4_S) was obtained from Sigma-Aldrich. For the trapping experiments, isopropanol and benzoquinone were obtained from Acros, and ethylenediaminetetraacetic acid disodium salt dihydrate (EDTA) was purchased from Fluka.

### Instrumentation

The CuIn_x_S_y_ and ZnS@CuIn_x_S_y_ composites were synthesized using an Anton-Paar microwave reactor (Monowave 400, Austria). The structure and phase purity of the synthesized materials were analyzed using powder X-ray diffraction (XRD, MiniflexII; Rigaku, Japan). X-ray photoelectron spectroscopy (XPS) was performed using a Kratos Analytical, AXIS Nova to determine the chemical states and elemental compositions of the synthesized materials. Fourier transform infrared spectroscopy (FT-IR) was used to identify the vibrational modes present in the synthesized materials. The FT-IR spectra of the synthesized materials were recorded using an IRspirit Fourier transform infrared spectrometer (IRSpirit, Shimadzu, Japan) in the wavenumber range 400–4000 cm^−1^ using the KBr pellet method. Raman spectra were obtained using a JASCO NRS-5100 Micro Raman Spectrometer equipped with a 532.06 nm laser. The optical band gap energies of the materials were determined using ultraviolet–visible diffuse reflectance spectroscopy (UV–Vis DRS, Shimadzu UV-2600i, Japan). Further morphological and crystallographic information about the synthesized materials was obtained using field-emission transmission electron microscopy (FE-TEM) and selected area electron diffraction (SAED) conducted with a JEM-F200 (JEOL Ltd., Tokyo, Japan). The average pore size, pore volume, and BET surface area of CuIn_x_S_y_ and 30% ZnS@CuIn_x_S_y_ were measured using a surface area analyzer (Quantachrome autosorb-iQ, Austria). For photocatalytic studies of the synthesized materials, the experiments were carried out in a Toption (TOPT-V) photochemical reactor with a 300 W Xe lamp as the UV–visible light source (wavelength > 350 nm). The intensity of the light at the location of the reaction vessel was ~ 14 mW/cm^2^, as measured by a Thorlabs PM100D power meter with a S401C thermal sensor. A Shimadzu UV-1900 UV–visible spectrophotometer was utilized to monitor the absorbance of BG and 4-NP in the spectral range 200–800 nm. A general-purpose pH meter (GP353 EDT direction, United Kingdom) was used to determine the pH of the 4-NP aqueous solution.

### Synthesis of CuIn_x_S_y_

CuIn_x_S_y_ was synthesized using a simple microwave-assisted synthesis method as reported in a previous study^28^. In a typical synthesis, 0.2416 g of Cu(NO_3_)_2_·3H_2_O, 0.3008 g of In(NO_3_)_3_, and 0.3045 g of thiourea were added into 15 mL ethylene glycol and the mixture was loaded into a 30 mL quartz vessel (G30 vial). The vessel was purged with N_2_ gas and sealed with a septum then rapidly heated to 200 °C by 850 W microwave irradiation for 10 min with continuous stirring to ensure homogenous heating. After the resulting product was cooled to ambient temperature, it was washed three times with distilled water and ethanol and collected by centrifugation (3500 rpm, 5 min per wash). Finally, the product was dried at 80 °C for 4 h to yield a black powder. Although pure, stoichiometric CuInS_2_ was the intended synthesis product, the formation of a CuS secondary phase is almost inevitable; therefore, we use the term CuIn_x_S_y_ throughout the manuscript to simplify the notation.

### Synthesis of ZnS@CuIn_x_S_y_ composites

For the synthesis of ZnS@CuIn_x_S_y_ composites containing different weight percentages (%) of ZnS, the procedure is similar to that mentioned above. In brief, a certain amount of Zn(CH_3_COO)_2_·H_2_O and thiourea were added into a suspension of 0.2 g of CuIn_x_S_y_. The mixtures were purged with N_2_ gas and heated to 200 °C by 850 W microwave irradiation for 10 min with continuous stirring. The obtained products were washed three times with distilled water and ethanol, dried at 80 °C for 4 h, and labelled as 10% ZnS@CuIn_x_S_y_, 20% ZnS@CuIn_x_S_y_, and 30% ZnS@CuIn_x_S_y_.

### Photocatalytic degradation of brilliant green dye

The photocatalytic activity of the as-prepared materials was evaluated by degrading BG dye at room temperature under visible light irradiation. All experiments were carried out in triplicate. A typical process was as follows: 10 mg of synthesized material was dispersed into 50 mL of 10 ppm BG aqueous solution. Then, the suspension was sonicated for 3 min, and it was later kept in the dark for another 3 min with constant stirring to reach an adsorption–desorption equilibrium of BG dye on the surface of the synthesized materials. The suspension was then irradiated with visible light and 3 mL aliquots were collected at intervals of 60 min for 5 h and transferred into centrifuge tubes to separate the photocatalyst from the dye pollutant solution. The aliquots were analyzed using a UV–Vis spectrophotometer in the range 200–800 nm and the photocatalytic activities of the pristine CuIn_x_S_y_, 10% ZnS@CuIn_x_S_y_, 20% ZnS@CuIn_x_S_y_, and 30% ZnS@CuIn_x_S_y_ were estimated by measuring the percentage of dye degradation using the following relation:

1 $$\text{\% degradation}=\frac{{A}_{0}-{A}_{t}}{{A}_{0}}\times 100$$

where *A*_0_ denotes the initial absorbance (the absorbance at time *t* = 0 h) and *A*_*t*_ denotes the absorbance of the aqueous dye solution after time *t* of treatment. Both *A*_0_ and *A*_*t*_ are recorded at the absorbance maximum (*λ*_max_) of the aqueous BG dye at 620 nm.

### Photocatalytic conversion of 4-NP

For the photocatalytic conversion of 4-NP, 10 mg of synthesized material was added and sonicated in 50 mL of 10 ppm 4-NP solution. After 3 min of adsorption/desorption equilibrium, the photocatalytic 4-NP conversion was initiated by irradiating the reaction mixture with a 300 W Xenon lamp. The activity was monitored using a UV–vis spectrophotometer. At fixed intervals of 1 h, 3 mL of aliquots were collected and filtered by centrifugation to separate the photocatalyst before analysis of the solution.

### Active species trapping experiments

To further investigate the main reactive species responsible for the photocatalytic degradation of BG using 30% ZnS@CuIn_x_S_y_ composite, trapping experiments were carried out in the presence of three typical trapping agents: isopropanol, benzoquinone, and disodium ethylenediaminetetraacetate (EDTA-2Na), which are utilized as scavengers of ^•^OH, O_2_^•–^, and h^+^, respectively. For 4-NP conversion, H_2_O_2_ was used to trap e^−^ in addition to other trapping agents. These trapping agents were added to the aqueous BG dye solution/4-NP solution at the beginning of the photocatalytic reaction. This experiment was carried out under the same conditions used for the BG degradation and 4-NP conversion experiments.

## Results and discussions

### Powder X-ray diffraction analysis

**Figure 1:**
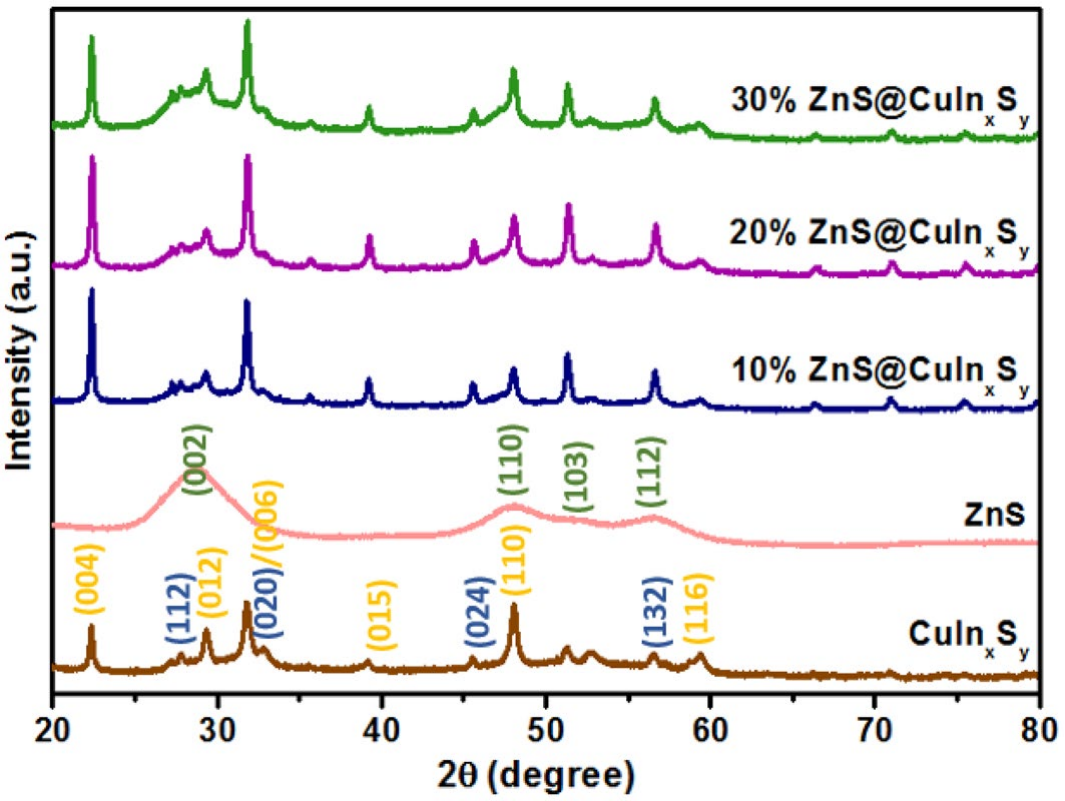
XRD patterns of CuIn_x_S_y_, ZnS, 10% ZnS@CuIn_x_S_y_, 20% ZnS@CuIn_x_S_y_, and 30% ZnS@CuIn_x_S_y_. The Miller indices given in blue, orange, and green indicate diffraction peaks arising from CuInS_2_, Cus, and ZnS, respectively.

Powder XRD was used to examine the crystal structures and phases of CuIn_x_S_y_, ZnS, and ZnS@CuIn_x_S_y_ composites with different percentages of ZnS (10%, 20%, and 30%), and the results are displayed in Fig. 1. The XRD pattern of CuIn_x_S_y_ indicated the formation of the tetragonal phase of CuInS_2_ (ICDD 98–060–0582: indexed in blue) along with the hexagonal phase of CuS (ICDD 98–006–7581: indexed in orange), as shown in Figure S1. This phenomenon suggests that Cu^2+^ ions could not be completely reduced by ethylene glycol^23^. The diffraction peaks of ZnS are well-matched with the reported data (ICDD 98–004–2793). The characteristic peaks located at 28.8°, 48.0°, 51.9°, and 56.5° can be observed clearly and corresponds to (002), (110), (103), and (112) of hexagonal ZnS (as indexed in green), respectively^38^. All of the synthesized ZnS@CuIn_x_S_y_ composites exhibited the main diffraction peaks of CuInS_2_, CuS, and ZnS. The successful loading of ZnS was confirmed by observation of the broad ZnS diffraction peaks around 28° and 48° superimposed on the CuInS_2_ and CuS diffraction patterns in the composites, as illustrated in Figure S2. Thus, XRD results confirmed the successful synthesis of ZnS@CuIn_x_S_y_ using microwave-assisted synthesis using ethylene glycol as the solvent.

### X-ray photoelectron spectroscopy

**Figure 2:**
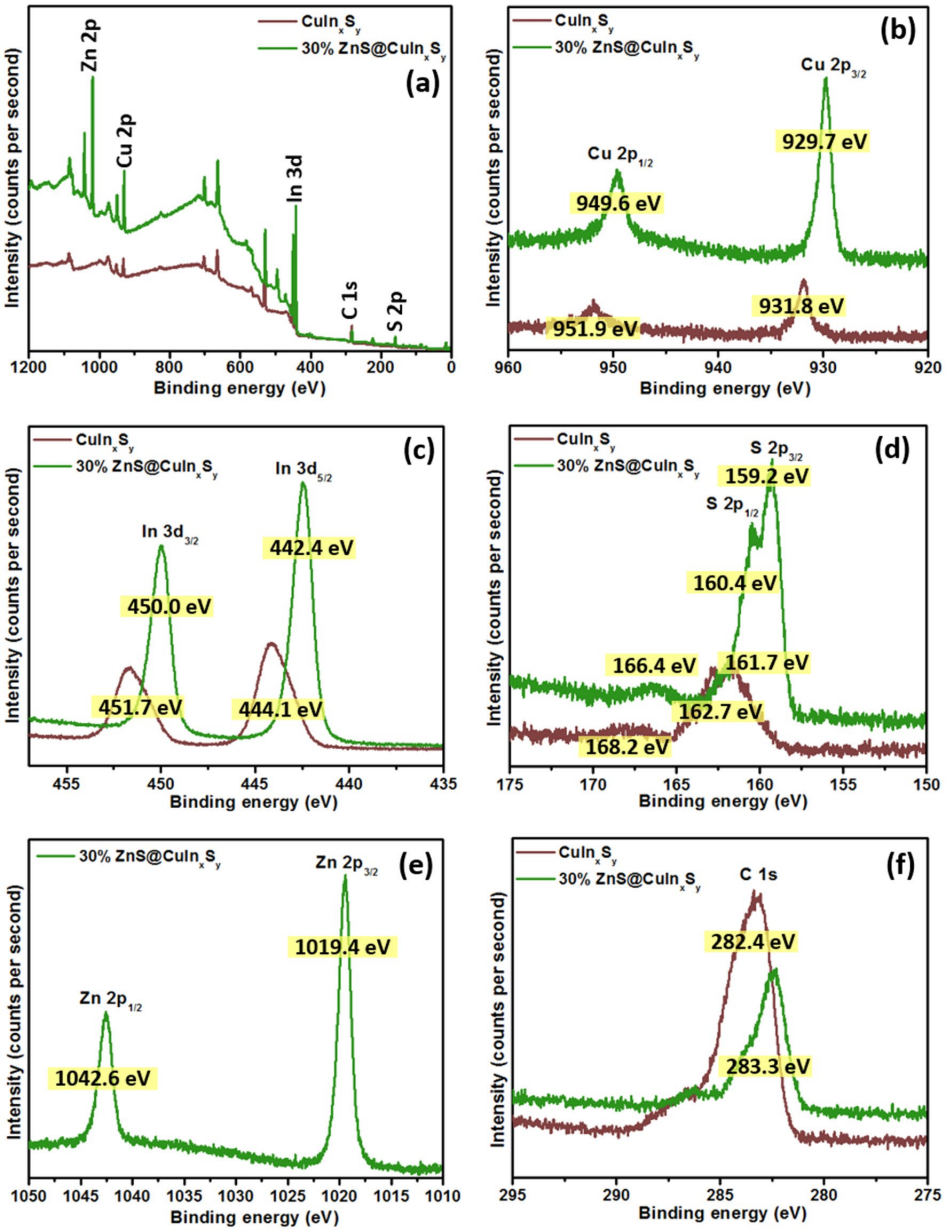
XPS spectra of CuIn_x_S_y_ and 30% ZnS@CuIn_x_S_y_: (**a**) survey scan, (**b**) Cu 2*p*, (**c**) In 3*d*, (**d**) S 2*p*, (**e**) Zn 2*p*, and (**f**) C 1*s*.

The chemical composition and oxidation states of the elements present in CuIn_x_S_y_ and 30% ZnS@CuIn_x_S_y_ were examined by XPS, as shown in Fig. 2. As depicted in the survey XPS (Fig. 2a), peaks corresponding to Cu 2*p*, In 3*d*, S 2*p,* and Zn 2*p* are observed clearly confirming the successful formation of CuIn_x_S_y_ and ZnS@CuIn_x_S_y_ composites. Figure 2b shows Cu 2*p* doublets at Cu 2*p*_3/2_ (931.8/929.7 eV) and Cu 2*p*_1/2_ (951.9/949.6 eV) for CuIn_x_S_y_ and 30% ZnS@CuIn_x_S_y_, respectively. The results are consistent with the presence of Cu(I), as expected for stoichiometric CuInS_2_, and in agreement with previous reports^32,39^. Moreover, the Cu 2*p* spectra showed no evidence of Cu^2+^ (in the form of Cu^2+^ ‘shake-up’ satellites around 944 and 965 eV)^40^ on the surface of both CuIn_x_S_y_ and 30% ZnS@CuIn_x_S_y_, which indicates that the CuS secondary phase observed in the XRD patterns is not present on the surface of the photocatalysts. The In 3*d* XPS spectrum in Fig. 2c exhibited two peaks at 444.1 eV, 451.7 eV for CuIn_x_S_y_ and 442.4 eV, 450.0 eV for 30% ZnS@CuIn_x_S_y_ which correspond to In 3*d*_5/2_ and In 3*d*_3/2_, respectively. This suggests that the oxidation state of In in the synthesized materials is + 3, as expected for CuInS_2_^29^. Figure 2d shows that the binding energies of S 2*p* in CuIn_x_S_y_ are at 162.7 and 161.7 belonging to S 2*p*_1/2_ and S 2*p*_3/2_, respectively and were separated by a spin–orbit splitting of 1.2 eV. Moreover, the peak positions of S 2*p* for 30% ZnS@CuIn_x_S_y_ are located at 160.4 and 159.2 eV^41^. The Zn 2*p* peaks of ZnS@CuIn_x_S_y_ as shown in Fig. 2e split into Zn 2*p*_3/2_ (1019.4 eV) and Zn 2*p*_1/2_ (1042.6 eV) can be assigned to Zn^2+^ with a peak separation of 23.2 eV^41^. The typical C 1*s* spectra shown in Fig. 2f arise from adventitious carbon. The XPS results also verify the successful synthesis of ZnS@CuIn_x_S_y_ composite via a microwave-assisted synthesis.

### Fourier transform infrared spectroscopy

**Figure 3:**
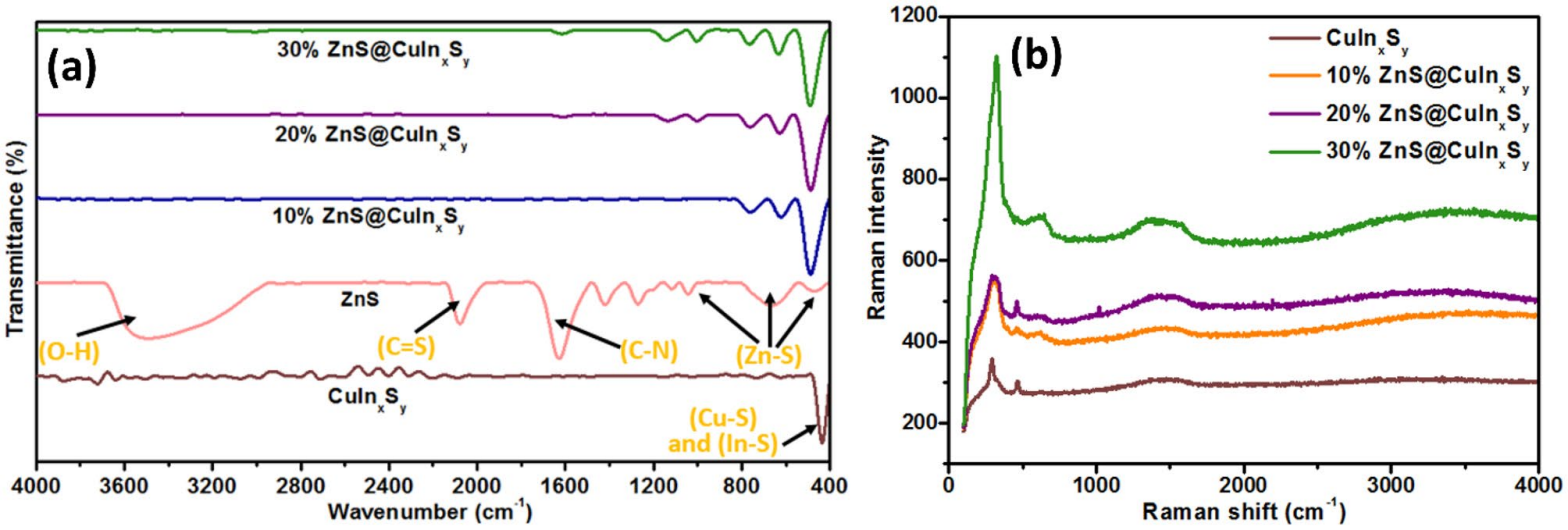
(**a**) FT-IR spectra and (**b**) Raman spectra of CuIn_x_S_y_, ZnS, 10% ZnS@CuIn_x_S_y_, 20% ZnS@CuIn_x_S_y,_ and 30% ZnS@CuIn_x_S_y_.

The vibrational modes present in the synthesized materials were determined via FT-IR. As shown in Fig. 3a, the band located at ~ 500–530 cm^−1^ can be assigned to the Cu–S^42^ and In–S^43^ stretching vibrations. Moreover, no other peaks can be observed in the FT-IR spectra, which reveals that all organic molecules from ethylene glycol and possible byproducts of the synthesis reaction were removed by washing with ethanol and distilled water^44,45^. For pure ZnS, the bands around 462 cm^−1^ and 667 cm^−1^ can be attributed to the characteristic Zn–S stretching vibration modes^46,47^. While, the bands around 1620 and 2070 cm^−1^ can be ascribed to the C–N and C=S vibrations of thiourea^48^. The FT-IR spectra of 10% ZnS@CuIn_x_S_y_, 20% ZnS@CuIn_x_S_y_, and 30% ZnS@CuIn_x_S_y_ exhibited similar Cu–S, In–S, and Zn–S bands. This confirms the presence of both CuIn_x_S_y_ and ZnS. Thus, FTIR measurements also verify the successful loading of ZnS onto CuIn_x_S_y_.

### Raman spectroscopy

Raman spectroscopy was employed to study the structure and bonding in CuIn_x_S_y_, ZnS, 10% ZnS@CuIn_x_S_y_, 20% ZnS@CuIn_x_S_y,_ and 30% ZnS@CuIn_x_S_y_. Figure 3b shows the Raman scattering spectra of CuIn_x_S_y_ and ZnS@CuIn_x_S_y_ composites with different ZnS contents. The Raman features broaden and shift continuously upward upon increasing ZnS content from 10 to 30%. The Raman spectrum of CuIn_x_S_y_ exhibited a strong peak at 290 cm^−1^ which may be assigned to the A_1_ mode of the chalcopyrite CuInS_2_^49^. A weak peak at 470 cm^−1^ may be assigned to the S–S stretching vibration mode of hexagonal CuS^50,51^. Moreover, no detectable peaks were observed for ZnS. Thus, the Raman spectra can only confirm the presence of CuInS_2_ and a secondary phase of CuS, which is in agreement with the XRD results.

### UV–visible diffuse reflectance spectroscopy

**Figure 4:**
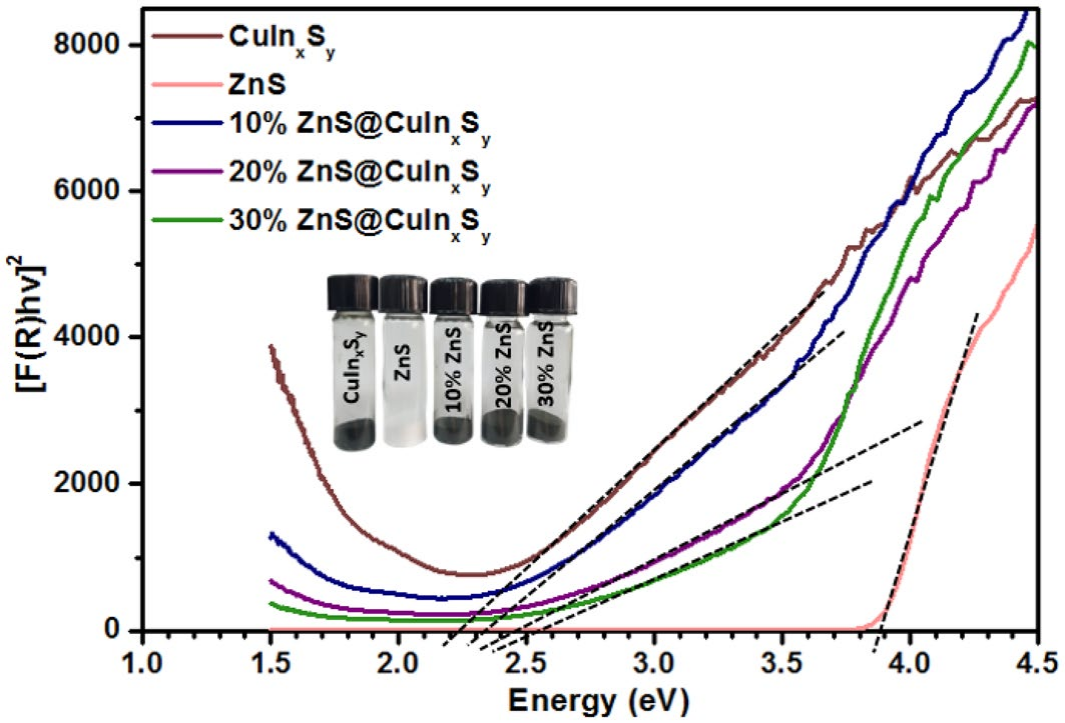
Tauc plots constructed from Kubelka–Munk transformed diffuse reflectance spectra for CuIn_x_S_y_, ZnS, 10% ZnS@CuIn_x_S_y_, 20% ZnS@CuIn_x_S_y,_ and 30% ZnS@CuIn_x_S_y_.

The effective band gap energies of the synthesized materials were determined by constructing Tauc plots from Kubelka–Munk transformed diffuse reflectance data, as shown in Fig. 4. The inset of Fig. 4 shows the photographic of synthesized CuIn_x_S_y_, ZnS, 10% ZnS@CuIn_x_S_y_, 20% ZnS@CuIn_x_S_y_, and 30% ZnS@CuIn_x_S_y_. The Kubelka–Munk function was used to estimate the effective band gap energy. The estimated band gap energy of CuIn_x_S_y_ was found to be 2.23 eV. While, for pure ZnS, the Tauc plot reveals a band gap energy of 3.87 eV, which corresponds to the sharp absorption onset at ~ 323 nm. As the ZnS content increases, the band gap energy of CuIn_x_S_y_ gradually increases from 2.23 to 2.51 eV. The effective band gaps of 10% ZnS@CuIn_x_S_y_, 20% ZnS@CuIn_x_S_y_, and 30% ZnS@CuIn_x_S_y_ were estimated to be 2.32, 2.41, and 2.51 eV respectively. The ZnS@CuIn_x_S_y_ composites exhibit narrow band gaps, which enable the materials to harvest visible light efficiently.

### Transmission electron microscopy

**Figure 5:**
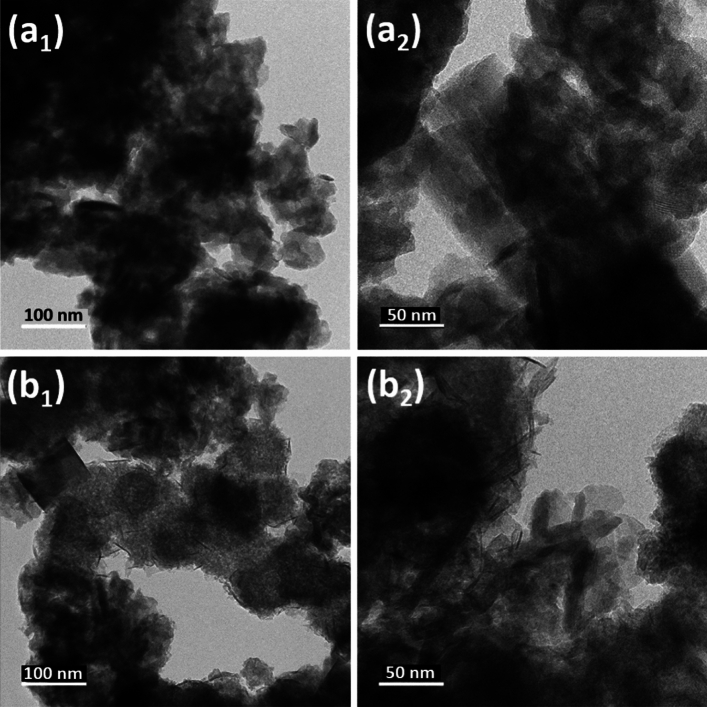
TEM images of (**a**_**1**_ and** a**_**2**_) CuIn_x_S_y_ and (**b**_**1**_ and **b**_**2**_) 30% ZnS@CuIn_x_S_y_ at two different magnifications.

The TEM images of CuIn_x_S_y_ and 30% ZnS@CuIn_x_S_y_ are shown in Fig. 5a and b, respectively. Figure 5a_1_ and a_2_ shows the rod-like structure of CuIn_x_S_y_ particles. While 30% ZnS@CuIn_x_S_y_ exhibited a needle-like structure, as shown in Fig. 5b_1_ and b_2_, which also shows that ZnS was well dispersed on the surface of the CuIn_x_S_y_ particles, and their close contact resulted in the formation of a ZnS@CuIn_x_S_y_ composite, which is helpful for fast interfacial charge carrier transfer. Thus, the photogenerated charge carriers can be utilized effectively, thereby enhancing the photocatalytic performance.

**Figure 6:**
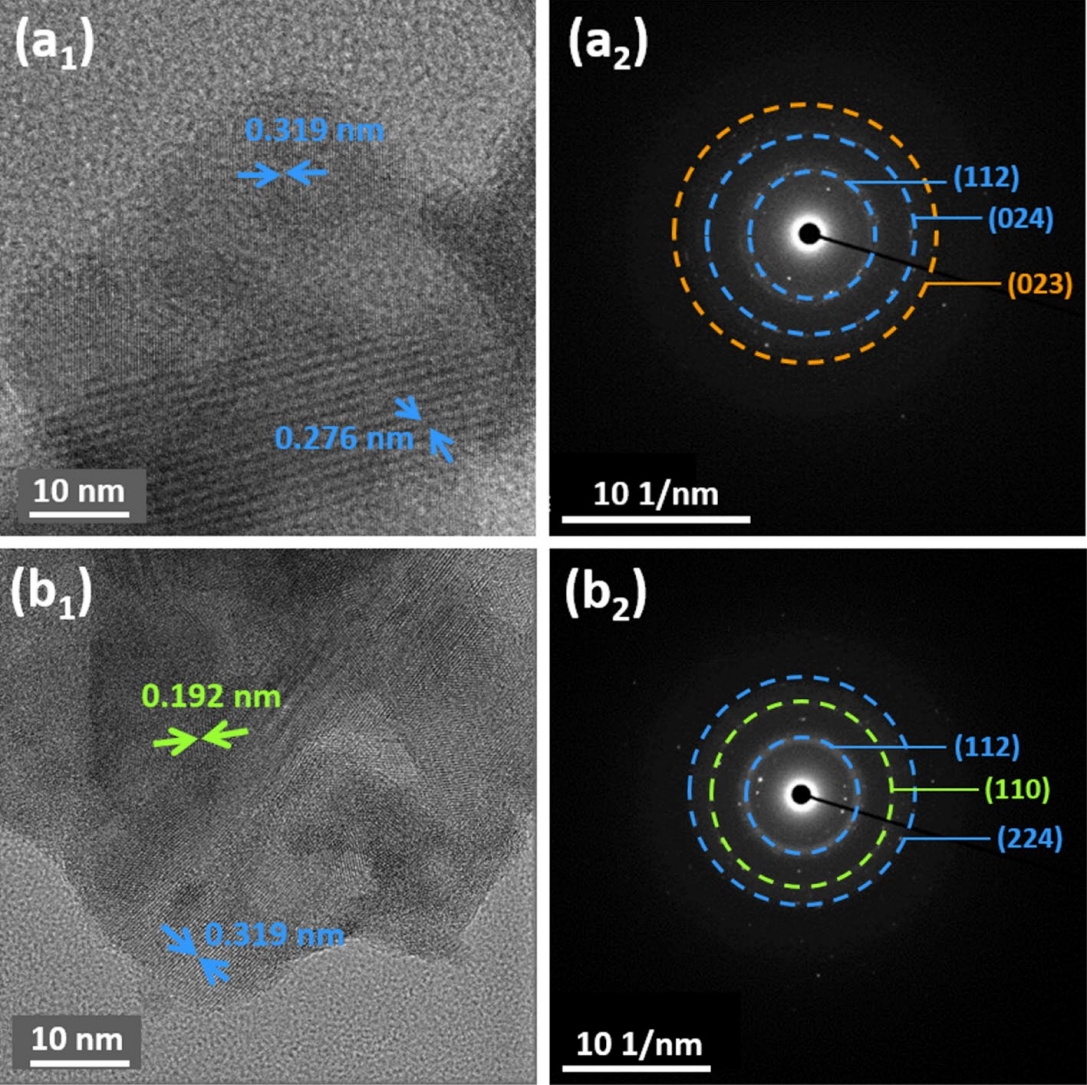
HR-TEM images and SAED patterns of (**a**_**1**_ and** a**_**2**_) CuIn_x_S_y_, and (**b**_**1**_ and **b**_**2**_) 30% ZnS@CuIn_x_S_y_ respectively. The Miller indices given in blue, orange, and green indicate the diffraction planes arising from CuInS_2_, Cus, and ZnS, respectively.

Figure 6a_1_ shows the HR-TEM image of CuIn_x_S_y_, the spacing of the lattice fringe of 0.276 and 0.319 nm matched well with (020) and (112) planes of tetragonal CuInS_2_, respectively. The 0.319 nm lattice fringe was also observed in Fig. 6b_1_, the HR-TEM image of ZnS@CuIn_x_S_y_ composites. The additional lattice fringe of 0.192 nm was assigned to the (110) plane of hexagonal ZnS. The SAED patterns of CuIn_x_S_y_ in Fig. 6a_2_ confirmed the presence of the (112) and (024) planes of tetragonal CuInS_2_ (indexed in blue) and the (023) plane of hexagonal CuS (indexed in orange). Moreover, the SAED patterns of 30% ZnS@CuIn_x_S_y_ as shown in Fig. 6b_2_ exhibit bright concentric rings corresponding to the (112) and (224) diffraction planes of tetragonal CuInS_2_ in addition to the (110) plane of hexagonal ZnS (indexed in green). These results are in accordance with the XRD results, which also confirm the successful loading of ZnS onto CuIn_x_S_y_.

### Brunauer–Emmett–Teller surface area analysis

**Figure 7:**
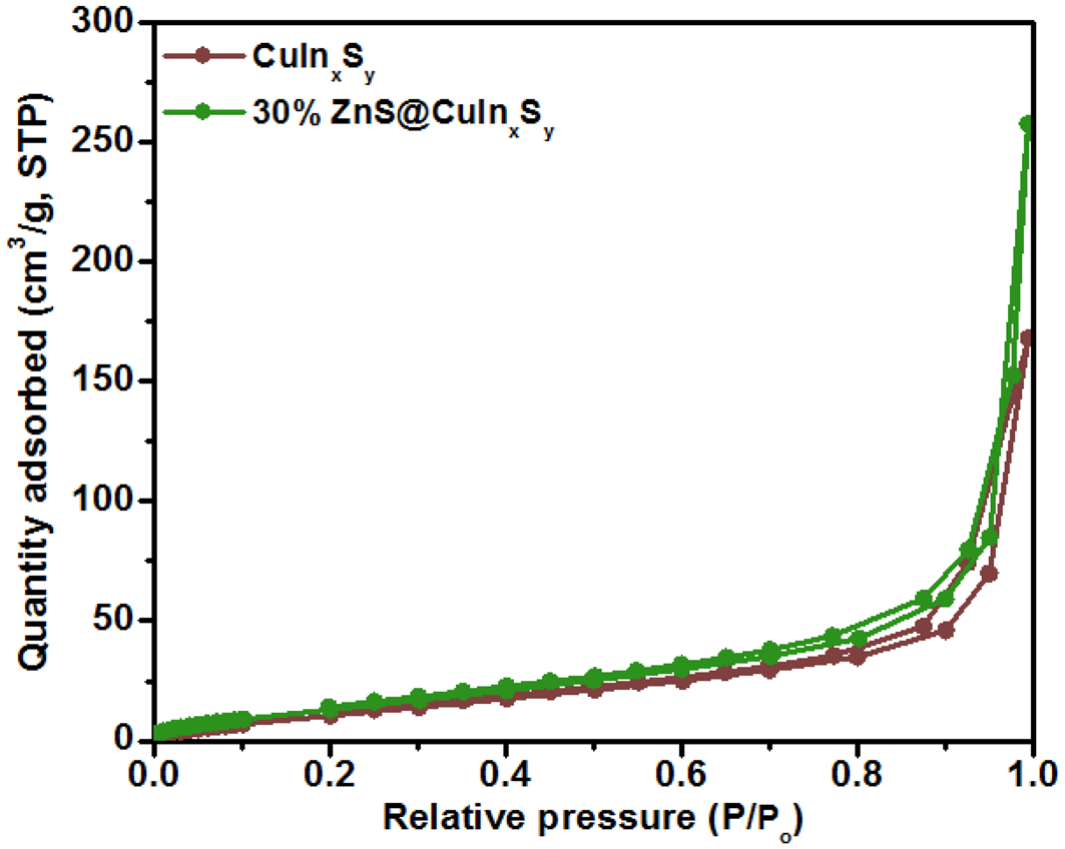
N_2_ adsorption and desorption isotherms of CuIn_x_S_y_ and 30% ZnS@CuIn_x_S_y_.

The BET N_2_ adsorption–desorption isotherm analysis was carried out to investigate the average pore size, pore volume, and BET surface area of CuIn_x_S_y_ and 30% ZnS@CuIn_x_S_y_. As shown in Fig. 7, the N_2_ adsorption/desorption isotherm of CuIn_x_S_y_ and 30% ZnS@CuIn_x_S_y_ revealed a type IV isotherm (according to the IUPAC classification) indicating the mesoporous feature of the materials. From Table 1, the CuIn_x_S_y_ exhibited a surface area of 46.9 m^2^/g, and with the introduction of 30% ZnS onto CuIn_x_S_y_, the surface area increased to 55.8 m^2^/g. The BET analysis revealed that the 30% ZnS@CuIn_x_S_y_ has a surface area with pore size and pore volume of approximately 142.7 Å and 0.398 cm^3^/g, respectively. This suggests that the addition of ZnS could provide more active surface sites. Thus, providing enough active sites for the adsorption of dyes on the surface of the photocatalyst and photocatalysis to take place.

**Table 1:** Average pore size, pore volume, and BET surface area of CuIn_x_S_y_ and 30% ZnS@CuIn_x_S_y_.

Synthesized material	Average pore size (Å)	Average pore volume (cm^3^/g)	Surface area (m^2^/g)
CuIn_x_S_y_	111.0	0.260	46.9
30% ZnS@CuIn_x_S_y_	142.7	0.398	55.8

## Photocatalytic activity of ZnS@CuIn_x_S_y_

### Photocatalytic degradation of brilliant green

**Figure 8:**
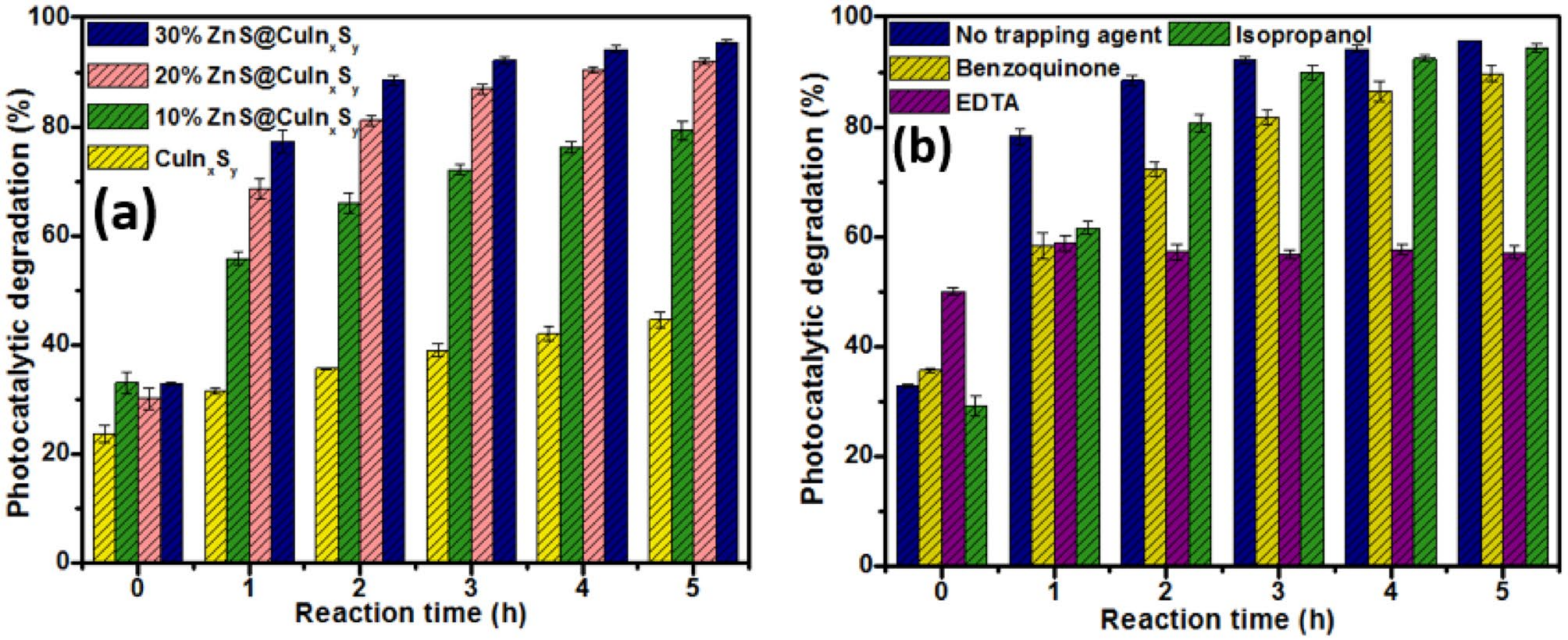
(**a**) Percentage photocatalytic degradation of BG using CuIn_x_S_y_, 10% ZnS@CuIn_x_S_y_, 20% ZnS@CuIn_x_S_y,_ and 30% ZnS@CuIn_x_S_y_ under visible light irradiation and (**b**) photocatalytic activity of 30% ZnS@CuIn_x_S_y_ with different trapping agents to determine the main reactive species responsible for the photocatalytic degradation of BG.

The photocatalytic efficiencies of CuIn_x_S_y_, 10% ZnS@CuIn_x_S_y_, 20% ZnS@CuIn_x_S_y,_ and 30% ZnS@CuIn_x_S_y_ for BG dye are presented in Fig. 8a. Prior to light irradiation, the dye and photocatalyst suspension were kept in the dark for 3 min under constant stirring to achieve an adsorption–desorption equilibrium. The percentages of BG adsorption were found to be 41.5% ± 3.96%, 53.7% ± 2.93%, 51.4% ± 1.05%, and 60.7% ± 2.06% for CuIn_x_S_y_, 10% ZnS@CuIn_x_S_y_, 20% ZnS@CuIn_x_S_y,_ and 30% ZnS@CuIn_x_S_y_, respectively, after 5 h in the dark, as shown in Figure S3. This shows that the CuIn_x_S_y_ and ZnS@CuIn_x_S_y_ composites have a high adsorptive affinity towards BG dye, which is crucial because if the dye molecules cannot be adsorbed on the surface of the synthesized materials, the photocatalytic activity would not be effective^52^. This may also be associated with the larger surface area, pore volume, and pore size of 30% ZnS@CuIn_x_S_y_ compared with CuIn_x_S_y_, as shown in Table 1. During the course of the photocatalytic reaction, the intensity of the BG dye solution gradually diminishes, and a gradual decrease in the absorption spectra of BG can be observed at the characteristic absorption peak height around 620 nm. Moreover, unmodified CuIn_x_S_y_ exhibited a poor photocatalytic performance of about 44.5% ± 1.36% under irradiation by visible light, which may be ascribed to the rapid recombination of charge carriers. The photocatalytic performance of CuIn_x_S_y_ is significantly enhanced as the amount of ZnS loaded increases from 10 to 30%. The photocatalytic activity of 30% ZnS@CuIn_x_S_y_ against BG was found to be considerably higher than those of unmodified CuIn_x_S_y_, 10% ZnS@CuIn_x_S_y_, and 20% ZnS@CuIn_x_S_y_. Within 5 h, 10% ZnS@CuIn_x_S_y_, 20% ZnS@CuIn_x_S_y,_ and 30% ZnS@CuIn_x_S_y_ were able to degrade about 79.2% ± 1.66%, 92.0% ± 0.50%, and 95.6% ± 0.08% of BG, respectively under visible light irradiation. The improved photocatalytic performance of 30% ZnS@CuIn_x_S_y_ is likely due to the presence of ZnS on the surface of CuIn_x_S_y_, which facilitates the separation and transfer of the photogenerated electrons and holes effectively resulting in superior degradation efficiency.

Three different scavengers including benzoquinone for O_2_^•–^, EDTA for h^+^, and isopropanol for ^•^OH were used to evaluate the main active species involved in the photocatalytic degradation of BG. As shown in Fig. 8b, the photocatalytic degradation of BG hardly decreased with the addition of isopropanol in comparison to other scavengers. Moreover, when benzoquinone was used to scavenge O_2_^•–^, a notable decrease in photocatalytic efficiency of 30% ZnS@CuIn_x_S_y_. The addition of EDTA showed the highest inhibition of the photocatalytic degradation of 30% ZnS@CuIn_x_S_y_ which confirmed the influence of h^+^ in the photocatalytic process.

### Photocatalytic conversion of 4-NP

**Figure 9:**
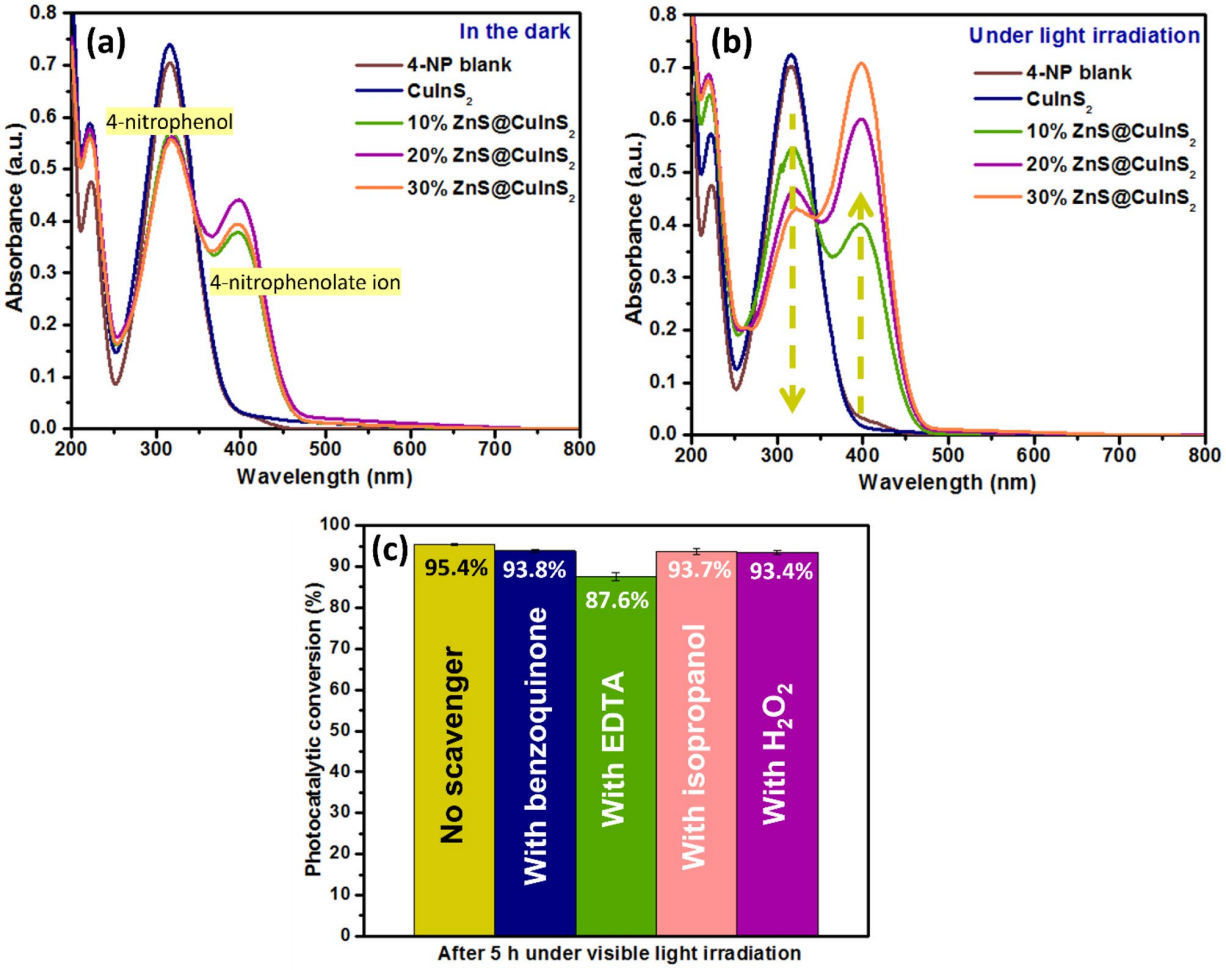
Photocatalytic conversion of 4-NP using CuIn_x_S_y_, 10% ZnS@CuIn_x_S_y_, 20% ZnS@CuIn_x_S_y,_ and 30% ZnS@CuIn_x_S_y_ (**a**) in the dark, (**b**) under visible light irradiation, and (**c**) photocatalytic activity of 30% ZnS@CuIn_x_S_y_ with different trapping agents to determine the main reactive species responsible for the photocatalytic conversion of 4-NP.

The photocatalytic conversion of 4-NP to the 4-nitrophenolate ion by CuIn_x_S_y_, 10% ZnS@CuIn_x_S_y_, 20% ZnS@CuIn_x_S_y,_ and 30% ZnS@CuIn_x_S_y_, as evidenced by UV–Vis absorbance spectroscopy, is shown in Fig. 9. In both the absence and presence of light, CuIn_x_S_y_ exhibited poor photocatalytic conversion activity in comparison to the other synthesized materials, as shown in Fig. 9a and b. Interestingly, when the ZnS@CuIn_x_S_y_ composites were added and irradiated with visible light, the maximum absorption peak of 4-NP shifted from 316 to 400 nm, and the colour of the solution changed from colourless to pale yellow due to the deprotonation of 4-NP to form the 4-nitrophenolate ion^53^. The conversion of 4-NP was gradually improved as the amount of ZnS loaded on the surface of CuIn_x_S_y_ increased from 10 to 30%. Among the synthesized materials, 30% ZnS@CuIn_x_S_y_ showed the highest conversion under irradiation by visible light. The active species involved in the process of 4-NP conversion were further identified by adding BQ, EDTA, isopropanol, and H_2_O_2_ to the solution mixture as scavengers to capture O_2_^•–^, h^+^, ^•^OH, and e^−^, respectively. Based on the reactive species trapping experiments (Fig. 9c), the addition of benzoquinone, isopropanol, and H_2_O_2_ resulted in equal inhibition of 4-NP conversion, which indicates that O_2_^•–^, ^•^OH, and e^−^ play equal roles in the conversion process. Moreover, the addition of EDTA to the mixture exhibited the highest inhibition of 4-NP conversion, which implies that photogenerated h^+^ are the main active species involved in the conversion process.

### Proposed photocatalytic mechanism

**Figure 10:**
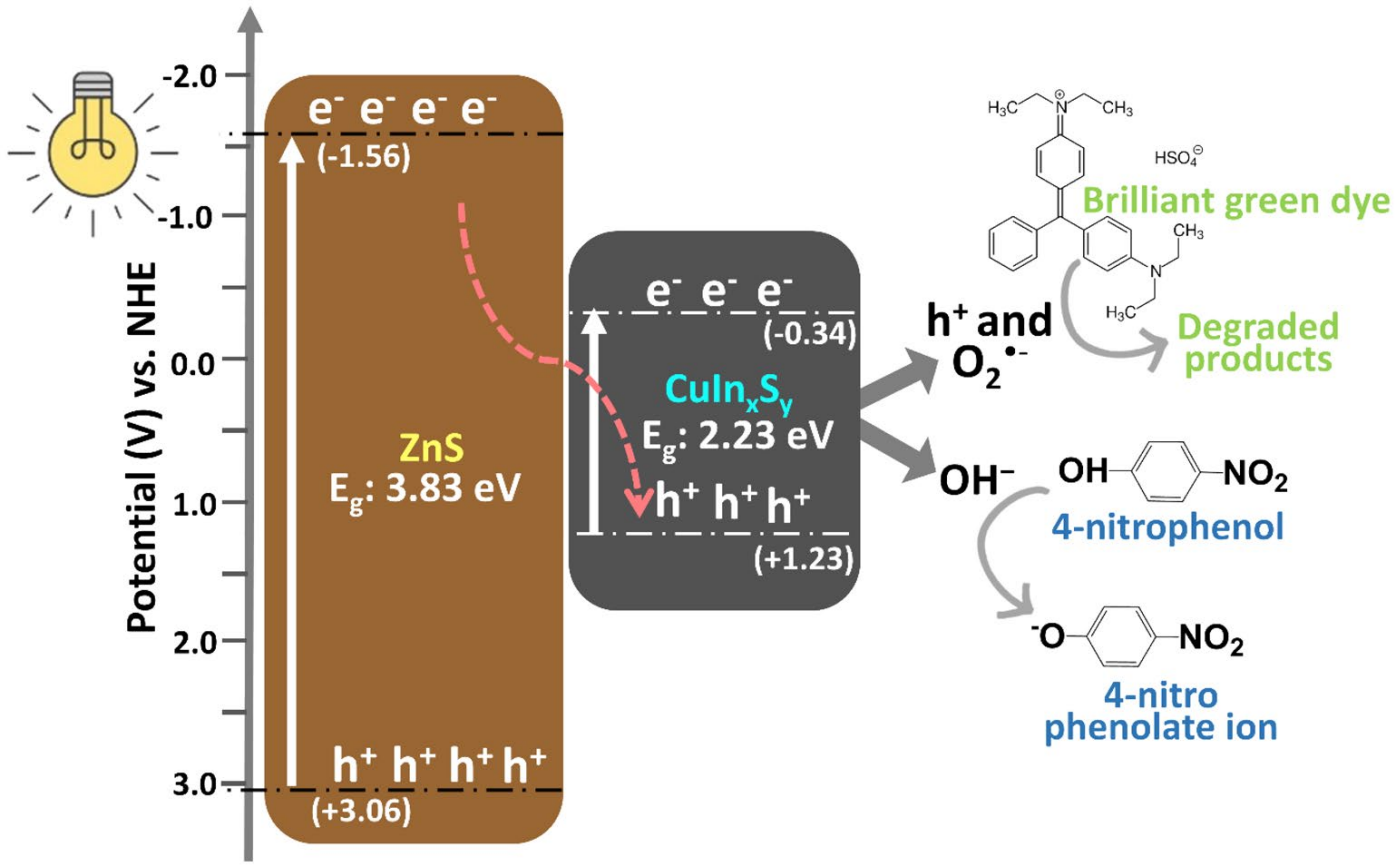
Proposed mechanism for the photocatalytic degradation of BG and conversion of 4-NP using ZnS@CuIn_x_S_y_.

The proposed photocatalytic mechanism of BG degradation using ZnS@CuIn_x_S_y_ is illustrated in Fig. 10 based on the band gap energy, the reported energies (specified as potentials vs. NHE) of the conduction band (CB) and valence band (VB) of ZnS and CuInS_2_, and the trapping experiments. The secondary CuS phase is not separately considered here because the XPS results indicate that it is not present at the surface of the photocatalyst particles. Based on the literature, CuIn_x_S_y_ is assumed to have *E*_CB_ = − 0.34 V and *E*_VB_ = 1.23 V^14^, while ZnS has *E*_CB_ = − 1.56 V and *E*_VB_ = 3.06 V^54^. Upon visible light irradiation, ZnS and CuIn_x_S_y_ simultaneously generate e^−^ and h^+^, in which photogenerated e^−^ are excited to their CB, leaving behind h^+^ in the VB. Next, the photogenerated e^−^ in the CB of ZnS are transferred to the VB of CuIn_x_S_y_, which improves the separation of photogenerated charge carriers and prolongs their lifetime. This also increases the probability of the photogenerated charge carriers participating in the redox reaction on the surface, which effectively improves the photocatalytic degradation performance. The photogenerated e^−^ in the CB of CuIn_x_S_y_ (*E*_CB_ = − 0.34 V) can reduce dissolved O_2_ to yield O_2_^•–^ (O_2_/O_2_^•–^, − 0.33 V vs. NHE), which agrees with the results of the active species trapping experiments. Alternatively, the photogenerated e^−^ can reduce the BG dye directly. Moreover, the photogenerated h^+^ of ZnS can react with H_2_O or OH^−^ species, oxidizing them into ^•^OH radicals, or the h^+^ can directly oxidize the BG dye to CO_2_ and H_2_O, which are harmless end products. Based on the active species trapping study, EDTA showed the highest inhibition followed by benzoquinone when compared to no scavenger, which indicates that h^+^ and O_2_^•–^ play important roles in the photocatalytic degradation process. Moreover, isopropanol shows the lowest inhibition, implying that ^•^OH is the least important species in the photocatalytic degradation of BG by ZnS@CuIn_x_S_y_.

In the case of the photocatalytic conversion of 4-NP, the enhanced conversion may be ascribed to the presence of OH^−^, which can either be formed through (i) direct reduction of H_2_O by e^−^_cb_, (ii) reduction of O_2_ to O_2_^•–^ and subsequent reaction with H_2_O to form H_2_O_2_ and OH^−^, or (iii) a two-step process in which H_2_O is first oxidized to ^•^OH by h^+^_vb_ and then e^−^_cb_ reduces ^•^OH to yield OH^−^ ions. It must be noted that in order for these reactions to result in a net increase in pH, some h^+^ must be consumed in a process other than water oxidation and/or H^+^ must be consumed by another process (e.g., conversion of 4-NP and/or 4-nitrophenolate ion to hydroquinone, as evidenced by the peak located ~ 225 nm in Fig. 9)^55^. The presence of OH^−^ increases the pH of the solution from 7.00 to slightly basic ~ 7.22, which deprotonates 4-NP to the 4-nitrophenolate ion. It is worth noting that the significance of the quite small pH change is dependent on the pK_a_ of 4-NP. Since the pK_a_ of 4-NP is 7.15^56^, this pH change is expected to cause a shift in the acid–base equilibrium from most of the 4-NP being protonated ([A^–^]/[HA] ≈ 0.71, according to the Henderson-Hasselbalch equation) to most being deprotonated ([A^–^]/[HA] ≈ 1.17), which is consistent with the observed changes in absorbance^57^.

## Conclusion

A series of highly efficient ZnS@CuIn_x_S_y_ nanocomposite photocatalysts with different ZnS loadings have been successfully synthesized via a microwave-assisted method and applied for the photocatalytic degradation of BG dye and 4-NP in aqueous solution. When compared to unmodified CuIn_x_S_y_, the composite materials showed enhanced photocatalytic performance under visible light irradiation, particularly 30% ZnS@CuIn_x_S_y_. Active species trapping experiments indicate that mainly h^+^ and O_2_^•–^ are involved in the photocatalytic processes. The formation of a heterojunction between CuIn_x_S_y_ and ZnS decreases photogenerated charge carrier recombination and thereby enhances the carrier separation efficiency in the composite. Therefore, this study provides a simple and effective route for the synthesis of a new visible-light active photocatalyst material and also highlights the importance of its application in the elimination of different organic contaminants from wastewater.

### Supplementary Information

Supplementary Figures.

## Data Availability

All data generated or analyzed during this study are included in the manuscript and SI.
