# Supplementary material for: Microwave-assisted synthesis of ZnS@CuInxSy for photocatalytic degradation of coloured and non-coloured pollutants
Source: Sci Rep. 2024 Jul 12;14:16155. doi: 10.1038/s41598-024-66100-2 (PMC11245618; doi:10.1038/s41598-024-66100-2)
Supplement: Supplementary file 1 — Supplementary Figures. [file 41598_2024_66100_MOESM1_ESM.pdf]

Supplementary material

Microwave-assisted synthesis of ZnS@$CuIn_xS_y$ for photocatalytic degradation of colored and non-colored pollutants

Ashmalina Rahman[1], Fazlurrahman Khan[2, 3, 4], James Robert Jennings[5,6], Young-Mog Kim[3, 4, 7], Mohammad Mansoob Khan[1,6]*

[1]Chemical Sciences, Faculty of Science, Universiti Brunei Darussalam, Jalan Tungku Link, Gadong, BE 1410, Brunei Darussalam.

[2]Institute of Fisheries Science, Pukyong National University. Busan 48513, Republic of Korea

[3]Marine Integrated Biomedical Technology Center, The National Key Research Institutes in Universities, Pukyong National University, Busan 48513, Republic of Korea.

[4]Research Center for Marine Integrated Bionics Technology, Pukyong National University, Busan 48513, Republic of Korea.

[5]Applied Physics, Faculty of Science, Universiti Brunei Darussalam, Jalan Tungku Link, Gadong, BE 1410, Brunei Darussalam.

[6]Optoelectronic Device Research Group, Universiti Brunei Darussalam, Jalan Tungku Link, Gadong, BE 1410, Brunei Darussalam.

[7]Department of Food Science and Technology, Pukyong National University, Busan 48513, Republic of Korea.

*Email: mmansoobkhan@yahoo.com and mansoob.khan@ubd.edu.bn

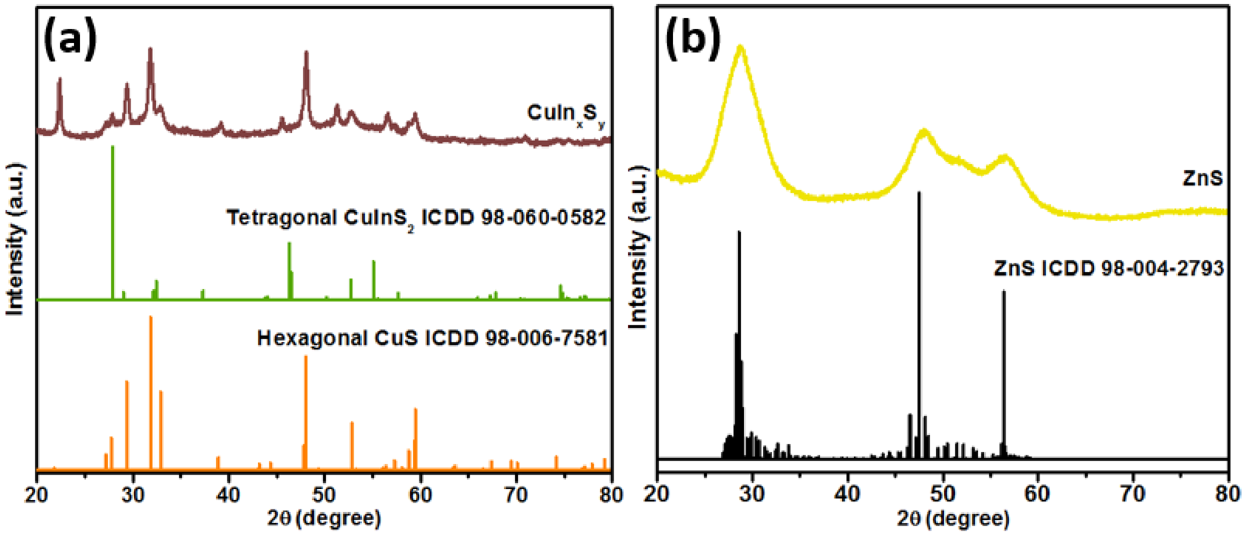

Figure S1. XRD patterns of **(a)** $CuIn_xS_y$ and **(b)** ZnS together with the relevant standards.

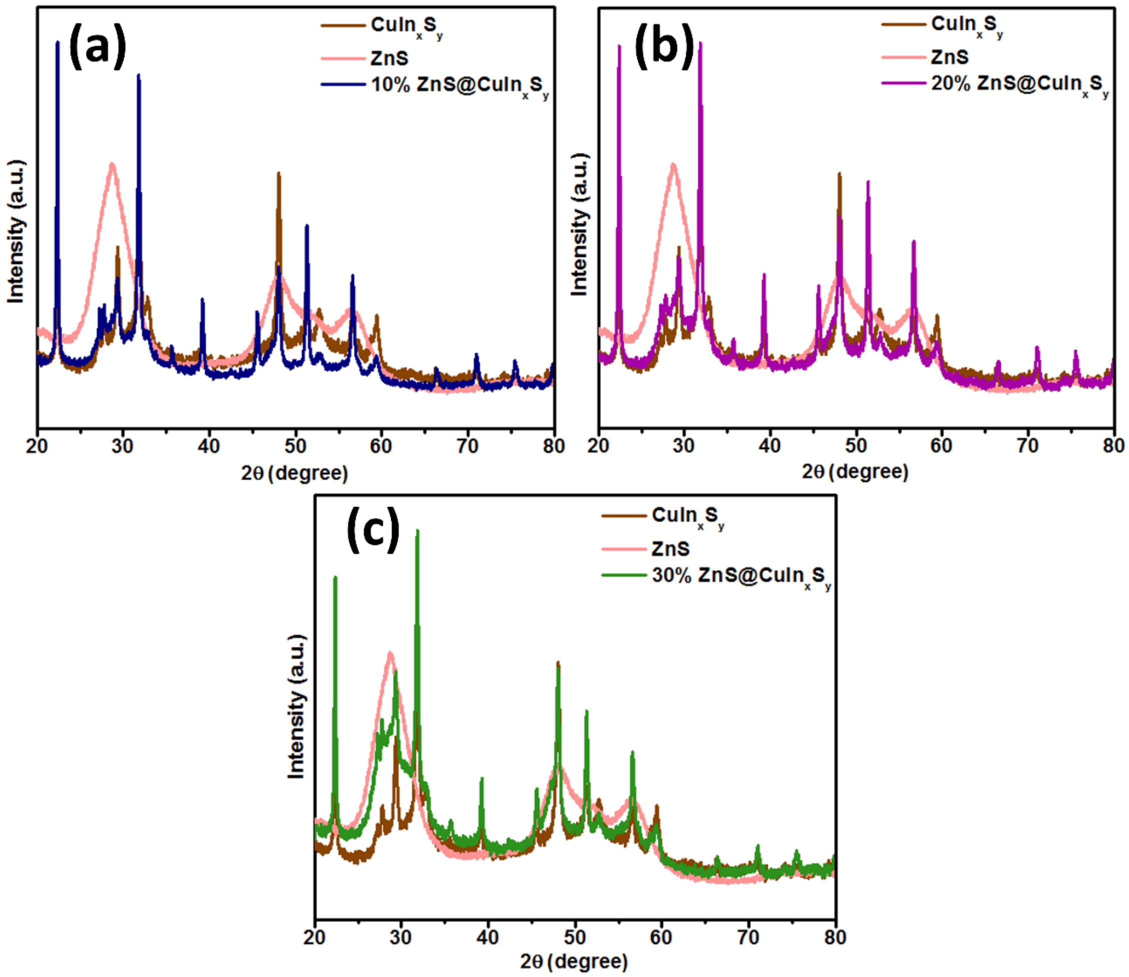

Figure S2. Overlapped XRD patterns of $CuIn_xS_y$ and ZnS with **(a)** 10% ZnS@$CuIn_xS_y$, **(b)** 20% ZnS@$CuIn_xS_y$, and **(c)** 30% ZnS@$CuIn_xS_y$.

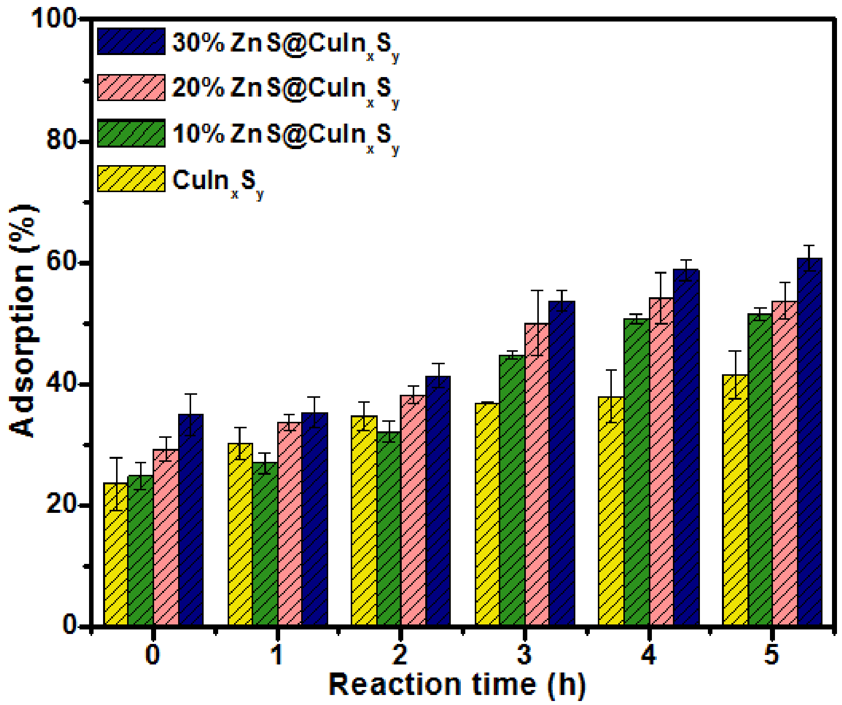

Figure S3. Adsorption of BG using $CuIn_xS_y$, 10% ZnS@$CuIn_xS_y$, 20% ZnS@$CuIn_xS_y$, and 30% ZnS@$CuIn_xS_y$ in the dark.
